# A Highly Miniaturized, Wireless Inertial Measurement Unit for Characterizing the Dynamics of Pitched Baseballs and Softballs

**DOI:** 10.3390/s120911933

**Published:** 2012-08-29

**Authors:** Ryan S. McGinnis, Noel C. Perkins

**Affiliations:** Mechanical Engineering, University of Michigan, 2350 Hayward St., Ann Arbor, MI 48105, USA; E-Mail: ncp@umich.edu

**Keywords:** inertial measurement unit, wireless sensor, baseball, softball, pitching, training

## Abstract

Baseball and softball pitch types are distinguished by the path and speed of the ball which, in turn, are determined by the angular velocity of the ball and the velocity of the ball center at the instant of release from the pitcher's hand. While radar guns and video-based motion capture (mocap) resolve ball speed, they provide little information about how the angular velocity of the ball and the velocity of the ball center develop and change during the throwing motion. Moreover, mocap requires measurements in a controlled lab environment and by a skilled technician. This study addresses these shortcomings by introducing a highly miniaturized, wireless inertial measurement unit (IMU) that is embedded in both baseballs and softballs. The resulting “ball-embedded” sensor resolves ball dynamics right on the field of play. Experimental results from ten pitches, five thrown by one softball pitcher and five by one baseball pitcher, demonstrate that this sensor technology can deduce the magnitude and direction of the ball's velocity at release to within 4.6% of measurements made using standard mocap. Moreover, the IMU directly measures the angular velocity of the ball, which further enables the analysis of different pitch types.

## 1. Introduction

Baseball and softball pitching demand highly dynamic full body movements with precise neuromuscular control. This control begins with the way the pitcher grips the ball, continues through the windup and delivery, and culminates in the ball's release. This instant in the sequence of the pitching motion is integral for the execution of different types of pitches; for instance, a fastball *versus* a breaking ball in baseball, or a rise ball *versus* a drop ball in fast pitch softball. Considerable research has addressed the flight path of the pitched ball after release and the governing aerodynamic forces [1–3]. Despite these advances, pitching coaches largely rely on qualitative assessments of pitching mechanics and outcomes in the form of visual inspection of the throwing motion, radar gun measurements, ball and strike counts, and ERA (earned run average) for training and skill assessment [4].

An important subset of studies focuses on “breaking” ball pitches, including the curveball [1–3]. The aerodynamic forces responsible for the “break” (or curve) in the flight path trace to the release conditions from the pitcher's hand including the orientation, spin, and velocity of the ball. In particular, experiments reveal that the total break of the flight path (1) is proportional to the ball's aerodynamic lift coefficient [2], (2) is dependent on the seam orientation [2], and (3) is a function of the magnitude and direction of the ball's angular velocity with respect to the velocity of its mass center [1]. The release conditions ultimately differentiate one pitch type from another. The fastball and changeup are released with substantial backspin in relation to the ball center velocity. By contrast, the curveball largely spins in the opposite direction; that is, it has substantial topspin. The slider has a combination of topspin and sidespin [5,6].

Studies of pitching mechanics largely rely on positional data obtained via high-speed cameras [4–11]. However, video-based motion capture is expensive, time consuming, and often requires measurements in the lab made by an operator skilled in both the collection and analysis of the data. Furthermore, baseball and softball angular velocity is very difficult to resolve using video based systems due to marker occlusion while the ball is in the pitcher's hand, and the high angular rates. Nevertheless, the angular velocity of the ball at release has an overriding influence on the subsequent ball flight path as noted above. For these reasons, it is quite challenging to use high speed motion capture to support pitcher training on the field of play.

The advent of MEMS inertial sensors and MEMS-scale wireless transceivers provide an attractive alternative to video-based motion capture for this application. Recent studies explore the use of wireless inertial measurement units (IMUs) for baseball pitcher training [12,13] among other sports training applications [14–18]. However, the size and mass of the IMUs employed in [12,13] (and those commercially available from companies like Xsens™, Culver City, CA, USA) preclude their use in measuring the motion of a baseball or softball.

To address these physical shortcomings, we introduce a highly miniaturized IMU that is directly embedded within the small confines of a baseball/softball. Doing so provides a low cost, highly portable and minimally intrusive technology for measuring the kinematics of a pitched ball right on the field of play. In particular, this technology provides a quantitative means for characterizing pitch type and consistency by resolving both the ball velocity and angular velocity at release, as well as throughout the pitching motion. We open this paper below with a description of the IMU hardware and the computational methods used to deduce ball-center velocity. We validate this method by benchmarking IMU-derived results with those obtained using a VICON (Los Angeles, CA, USA) motion capture system. In the process, we emphasize the probable ways that IMU-derived kinematical results can support pitcher training.

## 2. Sensor Design and Experimental Methods

**Figure 1.**
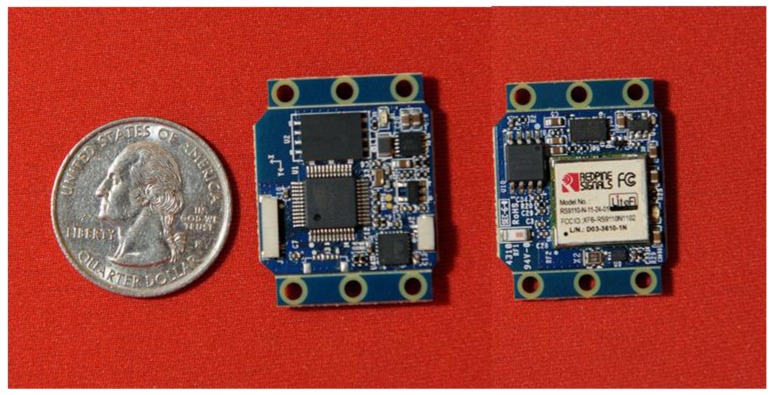
Highly miniaturized wireless IMU design used in this study was approximately the size of a quarter. The IMU provides three-axis sensing of acceleration and angular velocity with wireless data transmission to a host computer.

Figure 1 illustrates the IMU hardware employed in this study. This design was developed at the University of Michigan following a lineage of other multi- and single-board designs used for sports training, biomechanics, and rigid-body dynamics applications [14,15,17–19].

The design includes two sensing components. One is a digital tri-axial angular rate gyro, which performs internal 16-bit A/D conversion, with a measurement range of 2,000 deg/s, noise magnitude of 0.38 deg/s-rms for each axis (at 100 Hz output), and sampling frequency of 512 Hz. The other is a digital tri-axial accelerometer, which performs internal 13-bit A/D conversion, with a measurement range of 16 g, noise magnitude of 0.004 g-rms for each axis (at 100 Hz output), and sampling frequency of 800 Hz. Data from the IMU is low-pass filtered, with a cutoff frequency of 100 Hz before use. The IMU includes 8 Mbytes of onboard flash memory enabling operation in a data logging mode during trials after which the data can be downloaded to a host computer over a standard Wi-Fi network. The board measures a mere 30.1 × 23.7 × 5.1 mm and, when packaged with a switch and small lithium-ion battery, has a total mass of 12 g.

Prior to use, the IMU is calibrated following the procedure detailed in [16]. This procedure, which consists of two rotations about each of the three orthogonal sense axes, ultimately determines 24 calibration parameters (including scale factors, cross-axis sensitivity scale factors, and biases) for the IMU components. Doing so ensures that the acceleration and angular rate measurements are accurately resolved along a common orthogonal triad of sensor-fixed unit vectors. Bias values for the rate gyro are updated during each trial to ensure that changes due to temperature, battery voltage, or other external factors are captured.

**Figure 2.**
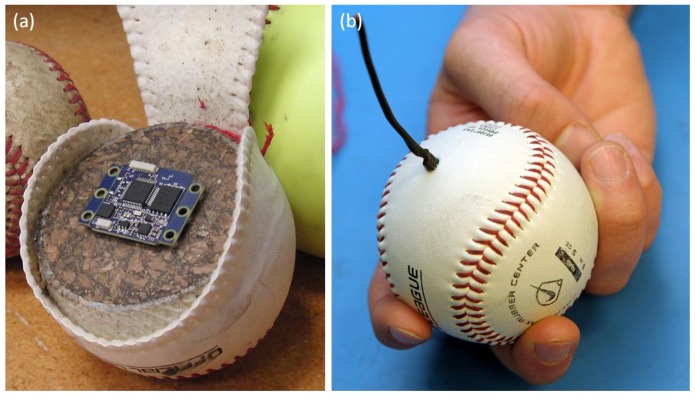
(**a**) The miniaturized IMU shown as it would be embedded in a baseball. (**b**) Final version of the ball including a small jack (switch/recharging) which is removed prior to the throw.

The IMU, battery, and switch are embedded in regulation softballs and baseballs enabling the measurement of ball dynamics during the throw. Figure 2 provides a “before” and “after” image of the instrumented baseball.

For both baseballs and softballs, the miniaturized IMU, battery, and switch are embedded in the ball (Figure 2(a)). Installation begins by unstitching half of the cover, after which the cork and rubber core is cut into two halves. Two small pockets are machined in the core, one to accommodate the battery and IMU and another for a switch which is accessible from the exterior via a hole in the cover large enough to accommodate a 2.5 mm phone plug. The IMU, battery, and switch are captured in these pockets with epoxy, and the two halves of the core are glued together prior to re-stitching the cover. The result is an instrumented baseball (Figure 2(b)) or softball. The mass of the instrumented baseball is within 0.1 g of its original (uninstrumented) mass (the softball is within 1.3 g). For either, this represents less than a 0.7% perturbation to the mass of the ball. For comparison, the official rules of baseball and softball dictate that the mass of a baseball may vary by as much as 7.1 g and the mass of a softball by as much as 20.4 g. It is important to mention that while the instrumented ball is able to survive repeated impacts with a catcher's mitt, it would not withstand an impact with a bat.

**Figure 3.**
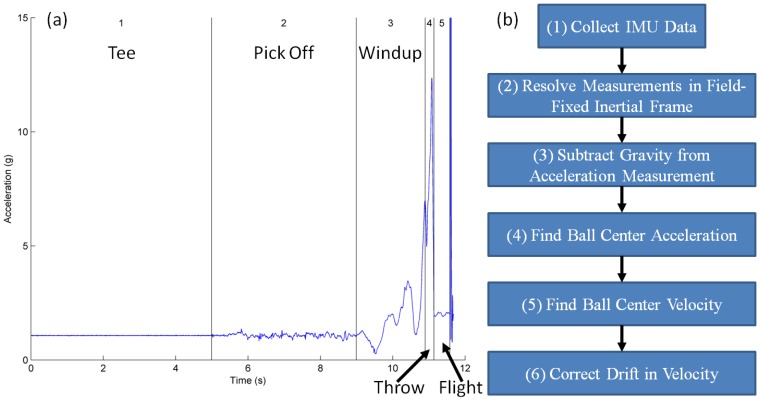
(**a**) Magnitude of acceleration as measured by the embedded IMU during a representative softball pitch. Major phases of the throwing motion are labeled 1–5. (**b**) Flow chart of major steps to calculate ball-center velocity during the throw.

As emphasized above, a major use of this technology is to measure the release conditions of the ball, that is, the velocity of the ball center and the angular velocity of the ball at release from the pitcher's hand. The angular velocity is measured directly from the angular rate gyro for pitches that remain within its measurement range. Unfortunately, the average MLB fastball rotates at about 15,000 deg/s which is considerably outside the range of the gyro used in this IMU [20]. Until recently, the best solution would be to estimate the angular velocity during free flight based on data from the tri-axial accelerometer following the methods discussed in [19]. However, subsequent to our study, MEMS device manufacturers (e.g., Analog Devices), released angular rate gyros capable of measuring angular rates up to 20,000 deg/s thus enabling application of the methods presented herein to potentially all baseball and softball pitches. In contrast, the velocity of the ball center must be computed (using data from both the gyro and the accelerometer) following the steps outlined in Figure 3(b) and as summarized below.

The following testing protocol was used. Each pitcher is instructed to pick the ball off of a tee, come to their natural “set” position, and then pitch the ball to the catcher. In addition, the subjects threw each pitch in a way to keep the ball angular velocity within the present measurement range of the gyro (2,000 deg/s). In this study data was collected from five pitches thrown by one baseball and one softball pitcher, both between 21 and 23 years of age. Both pitchers had previously competed at the high school level. Each pitcher was instructed to warm-up for as long as they determined was sufficient. They then threw 10 pitches to a catcher yielding five pitches with sufficient data from motion capture to enable benchmarking with this alternative measurement method. The vector magnitude of the measured acceleration for a representative softball pitch is reported in Figure 3(a), which also illustrates the major phases of the throwing motion.

Phase 1 corresponds to the time where the ball is in the tee prior to the throw. Phase 2 extends from the time where the pitcher picks the ball off of the tee to the instant she begins her windup (end of “set position”). Phase 3 begins at the start of the windup and extends to the instant when the pitcher's hand is furthest away from the catcher prior to release. Phase 4 extends from the end of Phase 3 to release. Finally, Phase 5 is the free-flight phase of the ball en-route to the catcher. The measured acceleration shown in Figure 3(a), combined with the measured angular velocity enable the calculation of ball-center velocity according to the steps outlined in Figure 3(b) and summarized next.

**Figure 4.**
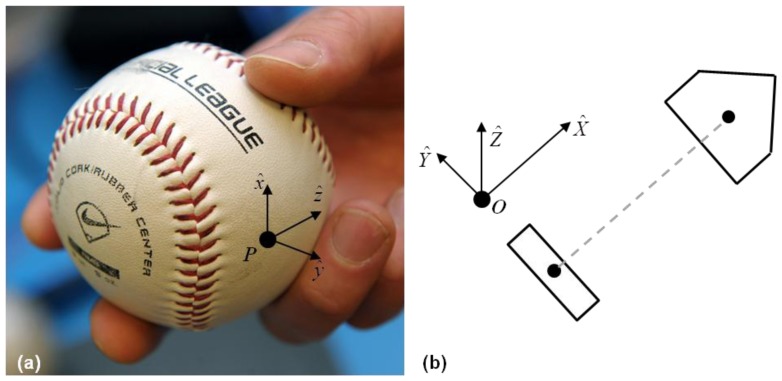
(**a**) Ball-fixed reference frame with origin at the center of the accelerometer (*P*). (**b**) Field-fixed reference frame with origin at location of the ball center in tee (*O*) at the start of the trial.

The acceleration and angular velocity data, measured in the non-inertial or “ball-fixed” frame, are transmitted to and stored on the host computer. This data must ultimately be resolved into components associated with an inertial (field-fixed) frame; refer to Step 2 in Figure 3(b) and to Figure 4. The ball-fixed reference frame is denoted by the mutually orthogonal triad of unit vectors (*x̂, ŷ, ẑ*) with origin p at the center of the accelerometer; refer to Figure 4(a). The inertial, or “field-fixed” frame is denoted by the triad of unit vectors (*X̂, Ŷ, Ẑ*) with origin located at the ball center when placed in the tee (point *O*; Figure 4(b)).

The transformation (direction cosine matrix) that relates these two frames and the differential equation which governs its evolution over time are:

(1) $$\vec{x}{|}_{\hat{X},\hat{Y},\hat{Z}}=\Lambda \vec{x}{|}_{\hat{x},\hat{y},\hat{z}}$$

(2) $$\dot{\Lambda }=\Lambda \hat{\vec{\omega }}$$

where Λ is the standard definition of a direction cosine matrix (DCM) [21], *x⃑*|*_x̂,ŷ,ẑ_* denotes the components of a vector *x⃑* resolved in the ball-fixed frame, and *x⃑*|*_x̂,ŷ,ẑ_* denotes the components of the same vector resolved in the field-fixed frame. During the throwing motion, the ball-fixed frame is both translating and rotating with respect to the field-fixed frame. As described below, the DCM is a function of the angular velocity of the ball, and is computed upon integrating (2) following an adaptation of the algorithm in [22]. The adapted algorithm employs a numerical approximation to (2) in which Λ̇ denotes the time derivative of the DCM and 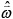 denotes the ball-fixed angular velocity vector in skew-symmetric form. The midpoint approximation to the solution of (2) yields the DCM at time step n+1 in terms of its value at time step n per:

(3) $$\Lambda_{n+1}=\Lambda_{n}\left[\text{I}+\frac{1}{2}\hat{\vec{\theta }}\right]\;{\left[\text{I}-\frac{1}{2}\hat{\vec{\theta }}\right]}^{-1}$$

In Equation (3), 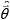 denotes the midpoint approximation of the change in orientation from time step n to n+1 in skew-symmetric form defined by:

(4) $$\hat{\vec{\theta }}=\frac{1}{2}\Delta t\left({\hat{\vec{\omega }}}_{n+1}+{\hat{\vec{\omega }}}_{n}\right)$$

where 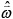
_*n*+1_ and 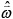
*_n_* are the (measured) ball-fixed angular velocities at time steps n+1 and n respectively in skew-symmetric form, and Δ(*t*) is the time interval between time steps n and n+1. Thus, the IMU provides the ball-fixed angular velocity needed to solve for the time-varying DCM, Λ(*t*), via Equation (3) provided an initial value, Λ(0), is also known. This initial value is determined by employing the accelerometer as an inclinometer during Phase 1 while the ball remains at rest in the tee following the procedure detailed in [15]. Thus, the acceleration vector can now be resolved in the field-fixed reference frame completing Step 2 in Figure 3(b).

Since the accelerometer measures signals down to 0 Hz, the resulting measurement also includes the acceleration due to gravity which must now be subtracted. Removal of gravity and computation of the ball-center acceleration (Steps 3 and 4 in Figure 3(b)) follows from:

(5) $${\vec{a}}_{c}(t)=\Lambda (t)\left[{\vec{a}}_{m}(t)+\dot{\vec{\omega }}(t)\times {\vec{r}}_{c/p}+\vec{\omega }(t)\times (\vec{\omega }(t)\times {\vec{r}}_{c/p})\right]-g\hat{K}$$

in which *a⃑_c_* is the ball-center acceleration resolved in the field-fixed frame, *a⃑_m_* is the measured acceleration in the ball-fixed frame, *ω⃑* and 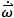 are the measured angular velocity and calculated angular acceleration (finite difference method), respectively, and *γ⃑_c/p_* is a position vector which locates the ball center relative to the center of the accelerometer. Direct integration of the ball-center acceleration (numerically using the trapezoidal method) yields the ball-center velocity per Step 5 of Figure 3(b) subject to the initial condition that this velocity starts from zero as the ball is held still in the tee.

It is well established that integration of the IMU-measured acceleration introduces significant error in the velocity due to drift [23,24]. This drift error is approximately identified and removed per Step 6 of Figure 3(b). We do so by splitting the throw into three parts: Part 1 spans Phases 1 and 2, Part 2 spans Phases 3 and 4, and Part 3 corresponds to Phase 5. Each Part is characterized by qualitatively distinct ball dynamics and thus distinct error correction. To this end, we introduce polynomial approximations to the drift error for each field-fixed component of velocity during each Part per:

(6) $$V_{j}(t)=V_{uc,j}(t)-\left(C0_{j}+C1_{j}t+C2_{j}t^{2}+C3_{j}t^{3}\right)\;\text{where}\;j=x,y,z$$

where *V_j_* and *V_uc,j_* are the corrected and uncorrected *j*-components of velocity respectively. The right-hand side of Equation (6) contains the *j*-component of the polynomial drift error function with constant coefficients *C*0-*C*3 defined separately for Parts 1, 2 and 3. In some sections the coefficients of the higher order terms (*i.e., C*2 and/or *C*3) are identically zero resulting in a lower-order drift error polynomial. Specifically, all three components have cubic drift error functions during Part 1. During Parts 2 and 3 the *Ŷ*-component is linear while the *X̂*- and *Ẑ*-components are quadratic in time. The zeroth order term in Equation (6) enforces continuity of the corrected velocity component across the parts. The remaining coefficients are found by simultaneous solution of known velocity and position constraints on the ball as summarized in Table 1 where the phases noted refer to those defined in Figure 3(a). The positions reported in Table 1 are the measured (or estimated) height of the ball center (*P_z_*) at the pitcher's set position (end of Phase 2), the height of the center of the strike zone (end of Phase 5), and the horizontal distance (Δ*x*_5_) the pitch is thrown. Also required are the acceleration due to gravity (*a_gravity_*), the acceleration due to air drag (*a_drag_*, estimated according to [1]) and the time duration of Phase 5 (Δ*t*_5_). These latter quantities are used to estimate the change in each of the three velocity components during Phase 5 (Δ*V*_x,5_, Δ*V*_y,5_, Δ*V*_z,5_).

The accuracy of the IMU-derived ball velocity is established by comparing it to that measured using a 10-camera high speed motion analysis system (VICON). The baseball/softball, with embedded IMU, was coated in reflective tape and its 3-D positions were measured by the VICON system at a frequency of 100 Hz and with calibrated position errors less than 0.25 mm. The ball's position data was low-pass filtered with a cutoff of 8.33 Hz and then numerically differentiated to determine the ball-center velocity as reported next.

## 3. Results and Discussion

We illustrate the promise of this sensor technology in this application by comparing ball-center velocities determined from IMU and motion capture (VICON) data. For experiments conducted on both baseball and softball pitching, we examine pitches thrown so that the angular rates do not exceed the measurement range of the IMU (2,000 deg/s). We open with an example that exposes the velocity drift error that is then corrected by the algorithm described above.

**Figure 5.**
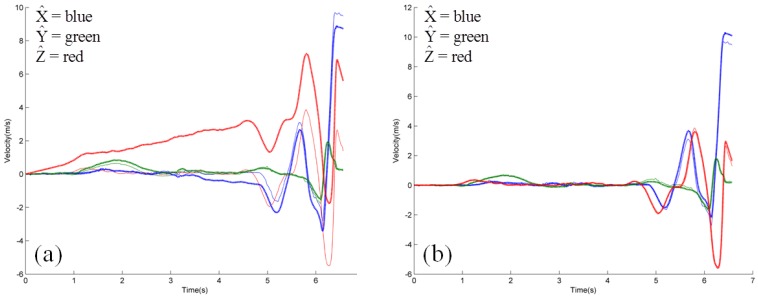
(**a**) Uncorrected and (**b**) Corrected ball-center velocity (m/s) components as determined by IMU (thick) and motion capture (thin) data for a representative softball pitch.

Figure 5(a) shows the ball-center velocity components in the field-fixed reference frame where the three field-fixed directions are distinguished as *X̂* = blue, *Ŷ* = green, and *Ẑ* = red. The thick lines designate the three uncorrected velocity components calculated from IMU data while the thin lines designate the same quantities calculated using motion capture data. Comparison of these two data sets reveals obvious drift error resulting from the integration of Equation (5). Figure 5(b) reports the same quantities but following the application of the drift error correction algorithm for the IMU data as described above. Inspection of this result shows excellent qualitative agreement between the IMU- and motion capture-derived results and in all major phases of the ball motion.

To now quantify this agreement, we introduce two metrics of the remaining small differences between the (drift corrected) velocity components based on the IMU data *versus* those measured using the motion capture data. The first metric is the normalized RMS difference between IMU-derived and motion capture-derived velocity components for the entire throwing motion:

(7) $$\varepsilon_{cj}=\frac{\sqrt{(1/N)\sum_{i=1}^{N}{\left(V_{cj,i}-{\tilde{V}}_{cj,i}\right)}^{2}}}{\left|\text{MAX}\left[V_{cj}(i\leq i_{\text{release}})\right]\right|},\;\text{for}\;j=x,y,z,$$

where *V_cj_* is the motion capture-derived *j*-component of velocity, *Ṽ_cj_* is the IMU-derived *j*-component of velocity, and *N* is the number of data samples. The numerator of Equation (7) is the RMS difference between the *j*-velocity components, while the denominator normalizes this difference based on the maximum value of the motion capture *j*-velocity component while the ball is in the pitcher's hand. The second error metric is the percent difference in the components of the *release* velocity with the motion capture-derived velocity components taken as the “truth data”:

(8) $$\varepsilon_{cj.\text{rel}}=\sqrt{\frac{{\left(V_{cj,\text{rel}}-{\tilde{V}}_{cj,\text{rel}}\right)}^{2}}{{V_{cj,\text{rel}}}^{2}}},\;\text{where}\;j=x,y,z$$

This second metric directly measures the accuracy of this sensor technology for determining the velocity of a pitched baseball/softball at release based on motion capture as the standard. A summary of these metrics for each velocity component, including mean and standard deviations, is reported in Table 2 for a sample of five baseball and five softball pitches.

The results of Table 2 for the first metric *ε_c_* demonstrate that the ball-center velocity obtained using the sensor technology recreates that obtained by standard motion capture to within 10% for baseball and 6% for softball over the entire range of the throwing motion. Considering the velocity of the ball-center at release, results for the second metric *ε_c,rel_* demonstrate agreement to within 4% for baseball and 4.6% for softball.

This latter result is particularly important given the overall influence of the release velocity (and angular velocity) in determining pitch type and quality. In understanding these comparisons, it is important to mention that the motion capture system, treated as yielding “truth data” here, is also susceptible to measurement errors. These errors in position largely arise from replacing data points lost due to marker occlusion and are then magnified when that position data is differentiated to determine velocity. Thus, the reported differences include contributions due to errors in the motion capture method as well.

**Figure 6.**
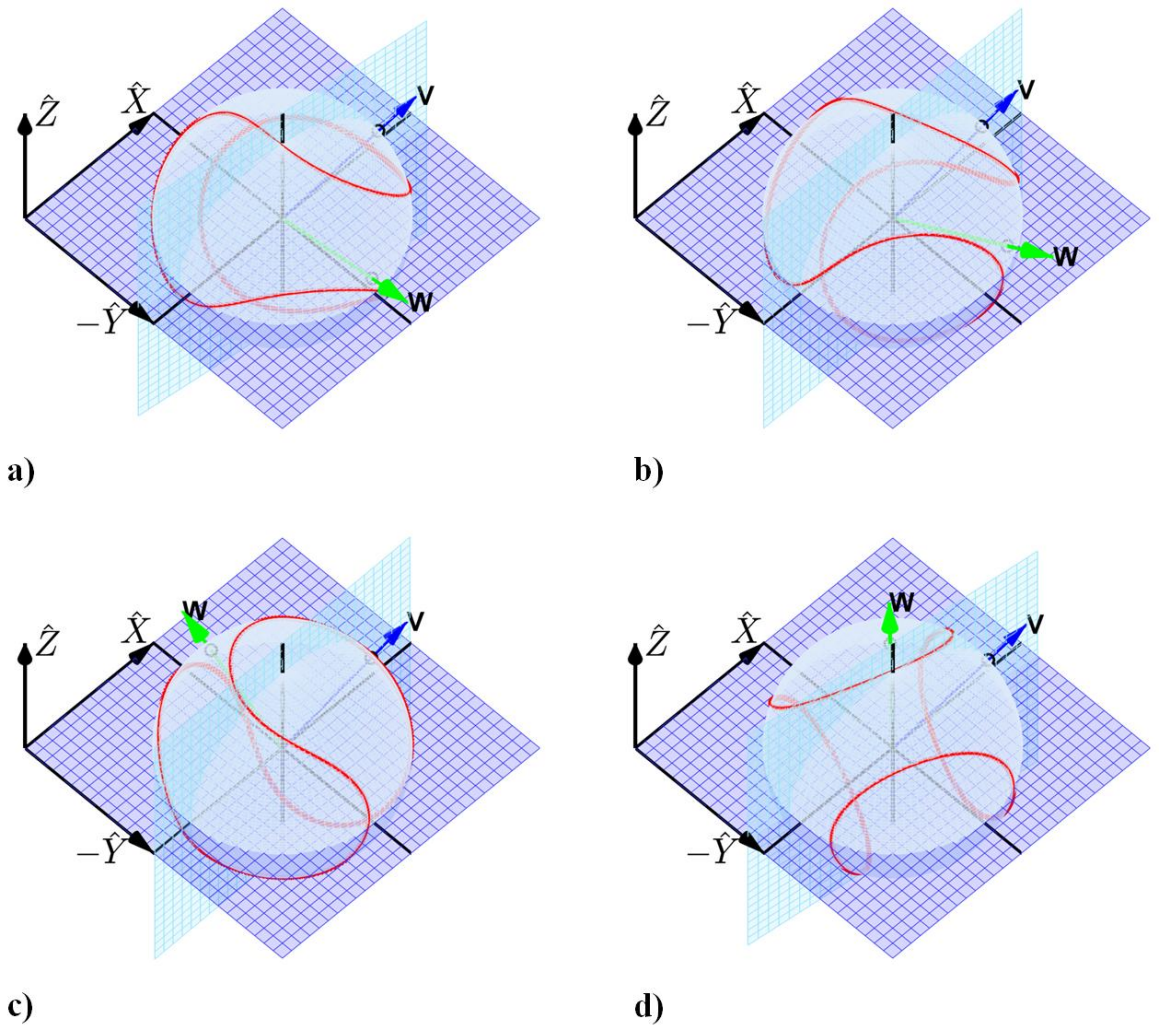
Linear (blue arrow denoted V) and angular (green arrow denoted W) velocity directions, and ball orientation at release for four typical baseball pitches: (**a**) fastball (four seam), (**b**) changeup, (**c**) curveball, and (**d**) slider.

As emphasized above, the sensor technology provides the data essential to resolving the release conditions of the ball including the ball orientation, ball center velocity (vector), and ball angular velocity (vector). These variables distinguish the types of pitches and they also provide a quantitative means for assessing the quality of each pitch type. By way of example, these potential uses are highlighted by the images of Figure 6 which illustrate the distinct release conditions for four common baseball pitches as measured by an embedded IMU (similar results were also obtained for the various types of softball pitches).

The images of baseball release conditions reported in Figure 6, for pitches thrown with modest linear and angular speed, confirm trends presented in [5,6]. Figure 6(a,b) illustrates the release conditions for a fastball and changeup, respectively. These two pitches are thrown largely with backspin which contributes to positive aerodynamic lift. This large backspin manifests as a large component of the angular velocity vector along the −*Ŷ* direction. Additionally, a small amount of lateral break develops due to the small but noticeable side spin component of the angular velocity; *i.e.*, the small component of *ω⃑* along the *Ẑ* direction. By contrast, Figure 6(c) shows that a curveball is released largely with top spin (note large component of *ω⃑* about *Ŷ*) which contributes to negative aerodynamic lift. Like the fastball and changeup, the small but readily visible side-spin component creates additional but small lateral break. Finally, Figure 5(d) shows the release conditions for a slider which is dominated by side spin (note large component of *ω⃑* about *Ẑ*) but also includes a small top spin component. The side spin induces a large lateral break, while the topspin induces a small drop. The position of the spin axis of the ball relative to the velocity of the ball center at release provides the essential information needed to evaluate whether the desired type of pitch is thrown correctly, to what degree the pitcher achieves that type of pitch, and (with multiple trials and measurements) how consistently it is being thrown. These measurement capabilities provide powerful information for evaluating pitching performance.

These results demonstrate the potential of a promising new sensor technology for use in baseball and softball pitcher training applications. However, it is important to emphasize the fact that most baseball and softball pitchers will generate ball angular velocities that exceed the measurement range of the MEMS angular rate gyros used in this study. Luckily MEMS sensor manufacturers have recently recognized the need for gyros with extended measurement ranges (20,000 deg/s) and these devices are now available (e.g., Analog Devices ADXRS649, Norwood, MA, USA) thus enabling the methods presented herein to be used for pitcher training at all levels of baseball and softball.

## 4. Conclusions/Outlook

The miniaturized wireless IMU technology presented herein has the potential to provide a low cost, highly portable measurement system to support pitcher training right on the field of play. The IMU-embedded baseball and softball faithfully reproduce the release velocity of the ball compared to measurements made by motion capture methods for the speed ranges considered. In particular, the difference between the IMU-derived release velocity and the motion capture-derived velocity remain less than 5%. Moreover, the IMU directly measures the angular velocity of the ball at release for pitches that remain within the measurement range of the angular rate sensors. Finally, subsequent to completion of this study, high range angular rate gyros have now entered the marketplace (e.g., Analog Devices ADXRS649). The velocity and angular velocity at release enable one to easily distinguish pitch types and the degree to which that pitch type is thrown. This quick visual and quantitative feedback will allow pitching coaches to accurately measure, and thereby improve, pitching performance.

## Figures and Tables

**Table 1.** Summary of velocity (V) and position (P) constraints used to determine drift correction polynomials in each of the three field-fixed directions.

*X̂*	*Ŷ*	*Ẑ*
*V_x_* = 0 during Phase 1	*V_y_* = 0 during Phase 1	*V_z_* = 0 during Phase 1
*V_x_* = 0 end Phase 2	*V_y_* = 0 end Phase 2	*V_z_* = 0 end Phase 2
*V_x_* = 0 end Phase 3	*V_y_* = 0 end Phase 4	*P_z_* = measured end Phase 2
*V_x_* = Δ*x*_5_/Δ*t*_5_ end Phase 4	Δ*V*_*y*,5_ = 0	*P_z_* = strike end Phase 5
Δ*V*_*x*,5_ = *a_drag_* Δ*t*_5_		Δ*V*_*z*,5_ = *a_gravity_* Δ*t*_5_

**Table 2.** Summary of differences, mean (standard deviation), between IMU- and motion capture-derived velocity components during the throwing motion and at the instant of release. All values are reported as a percentage.

	**Baseball**			**Softball**		

**Error**	*X̂*%	*Ŷ*%	*Ẑ*%	*X̂*%	*Ŷ*%	*Ẑ*%
*ε_c_*	2.5 (0.3)	10.0 (1.6)	7.7 (2.0)	2.2 (0.6)	5.9 (1.3)	3.3 (0.6)
*ε_c,rel_*	3.5 (2.4)	1.9 (2.1)	4.0 (3.6)	4.6 (2.3)	1.0 (0.5)	3.6 (1.3)
